# Supplementary material for: Are carbon emissions trading and green financial instruments synergistic? -Comprehensive quantitative research based on content analysis
Source: PLoS One. 2024 Mar 7;19(3):e0298601. doi: 10.1371/journal.pone.0298601 (PMC10919601; doi:10.1371/journal.pone.0298601)
Supplement: S1 Data — (ZIP) [file pone.0298601.s001.zip › Data/地级市Z_F工作报告.zip!/║┌┴·╜¡/┤≤╟∞╩╨/└┤╫╘╣1⁄2╓┌║┼í╛┬φ┐╦╩2╛▌═°í┐_2006.docx]

各位代表：

　　现在，我代表大庆市人民政府，向大会作政府工作报告，请予审议，并请市政协各位委员提出意见。

　　一、“十五”发展变化和2005年工作回顾

　　过去的五年，是我市经济发展速度最快的五年，社会事业全面提升的五年，城乡面貌变化巨大的五年，人民生活得到较多实惠，城市综合竞争实力显著增强。五年来，在市委的坚强领导下，我们迎难而上，励精图治，“十五”计划确定的经济社会发展主要目标和各项任务全面完成。

　　过去的五年，经济发展实现新跨越。地区生产总值由2000年的1029.4亿元增加到1400.7亿元，年均增长9.1%，超过“十五”计划目标2.1个百分点；地方财政总收入由2000年的33.2亿元增加到69.7亿元，年均增长16%；累计完成固定资产投资1160.4亿元，一大批产业和基础设施项目相继建成。中直石油石化工业稳步发展，地方工业高速增长，第三产业不断壮大。油与非油经济的比重，由2000年的77︰23调整到65︰35。

　　过去的五年，改革开放迈出新步伐。从支持大企业重组改制、接收企业办社会部分职能，到深化农村税费、国有企业、事业单位、财政管理体制和政府机构等一系列改革，经济社会发展活力明显增强。从完善软硬环境，到打造产业生态，招商引资取得丰硕成果，对外开放度不断提升。累计引进到位资金359.8亿元；实现进出口总额5.9亿美元，五年增长5倍。

　　过去的五年，城乡面貌发生新变化。累计投资387亿元，建设道路、桥梁、堤防和城市公共服务设施等1470项基础项目，城市功能不断改善；动迁改造棚户区75.8万平方米，建设经济适用住房145万平方米，整理老居住区128个，建设新住宅区491.8万平方米，荣获中国人居环境范例奖；推进生态市建设，植树造林，草原禁牧，治理湖泡，生态环境得到优化；实施“东移北扩”、“中联西拓”，城市空间不断扩大。大庆成为全国内陆首家环保模范城。

　　过去的五年，农村建设取得新成就。农业结构调整迈出重大步伐，畜牧业增加值占农业比重由2000年的38%提高到50%，奶牛存栏突破30万头。优质农产品基地规模不断扩大，抗旱保收田面积达到230万亩，标准化示范基地和绿色、有机、无公害食品基地发展到200万亩。农业产业化经营水平不断提升，市级以上农业产业化龙头企业达到38户。农村基础设施明显改善，建成通县乡村公路2985公里。积极贯彻党的惠农政策，扎实开展“一免两补”工作，为农民减轻税费负担和增加补贴2.3亿元。

　　过去的五年，城市综合竞争力得到新提升。实施大文化建设战略，科技、教育、卫生、文化、体育等社会事业长足发展。引进提升大庆石油学院、八一农大、哈医大大庆校、大庆师范学院等7所高校，引进建设东北林大大庆生物技术研究院、省化工研究院大庆分院、中加食品研究中心等5家科研院所。创建10所省级示范高中，改造建设247所中小学校。推进公共卫生体系建设，疾病预防控制工作成效明显。企业文化、社区文化、校园文化蓬勃发展，艺术精品不断涌现，以大庆精神、铁人精神为灵魂的大庆文化影响力进一步扩大。精神文明建设得到加强，大庆被评为全国创建文明城市工作先进城市。

　　过去的五年，人民生活水平又有新提高。在全国率先建立吃饭、住房、取暖、就业、就医、就学、法律援助“七位一体”综合救助体系。实行“五险”合一、地税征收，社会保险覆盖面进一步拓宽，社会保障体系更加完善。累计安置就业和再就业11.5万人次，城镇登记失业率控制在4.5%以内。社区功能不断完善，我市被评为全国社区建设示范城。城市居民人均可支配收入、农民人均纯收入比“九五”期末分别增加4569元和1629元，年均分别增长8.5%和20%。

　　各位代表，2005年是“十五”收尾之年，在市委正确领导下，在市人大、市政协的监督支持下，我们紧紧依靠全市人民，求真务实，锐意进取，着力抓好65件大事实事，圆满完成了市七届人大四次会议确定的发展目标和重点任务，以经济社会全面进步的喜人成绩，给“十五”画上圆满句号。

　　（一）经济保持快速增长。全市地区生产总值实现1400.7亿元，增长10%。地方财政总收入69.7亿元，增长3.1%。固定资产投资302.7亿元，增长26.6%。农业和农村经济发展创历史新高，实现增加值42.4亿元，增长11%。粮食总产236.9万吨、单产5407公斤/公顷，分别增长37.8%和34.8%。畜牧业实现增加值21.2亿元，增长28.8%。工业经济实力不断增强。石油工业稳步发展，生产原油4495万吨，实现增加值955.5亿元。石化工业规模扩大，60万吨乙烯改造、56万吨合成氨/76万吨尿素扩能、30万吨聚丙烯等重点项目竣工投产，规模以上石化工业实现增加值78亿元。地方工业快速增长，实现增加值130亿元，增长40.7%。其中，高新区实现工业增加值56亿元，增长40%。第三产业稳步发展，实现增加值154.8亿元，增长15.2%。社会消费品零售总额达到163.9亿元，增长19.2%，增幅全省第一。民营经济实现增加值193.9亿元，增长20.3%，占地方经济比重达到57.2%。旅游业总收入突破10亿元大关，创出最好水平。

　　（二）人民生活继续改善。城市居民人均可支配收入13662元，农民人均纯收入2723元，分别增长10.9%和10.7%。城乡居民储蓄存款余额526.6亿元，增长12.8%。居民消费价格总指数101.8%，城市恩格尔系数29.7%。新增就业3.8万人，转移农村劳动力7.6万人。参加养老、失业、医疗、工伤和生育保险人数增加9.9万人。城市低保金标准由143元提高到180元，发放政策性补助和基本生活费5100万元，7.3万人得到救助。完成红旗村和滨洲村2445户平房拆迁，建设经济适用住房和廉租廉价房23.5万平方米，解决4300户群众住房难问题。收缴企业农民工工资保障金2645万元，解决拖欠农民工工资1428万元。年人均收入低于1300元的51个贫困村、6000户实现脱贫。实施农村饮水安全工程120处，解决5.9万人饮水困难。通过价格补贴和减费，为市民和企业减负1.6亿元。将22所农村敬老院改造撤并成8所，新建社区老年文体活动站48个，新建和改建9所乡镇卫生院。

　　（三）城乡建设跨越发展。投资90多亿元，新建续建工程465个，建筑面积423万平方米，其中35项市级重点工程相继开工或建成，投资额、建设面积、竣工率和开工率均创历史新高。路桥建设实现历史性突破，g015大庆绕行线、经九街延伸如期推进，萨环东路立交桥、滨洲线7处“平改立”、让杜路建成通车，新建乡村公路956公里，超计划245公里。城市标志性建筑又添新亮点，教育文化中心、青少年科技活动中心、铁人纪念馆新馆等文体工程进展顺利，会议中心、行政服务中心工程破土动工。五湖新区拆迁12万平方米，50万平方米湖滨教师花园居住小区全面竣工。城市管理力度加大，拆除违法违章建筑24万平方米，改造38个老居住区环境，实现东风新村热源联网。城市经营收益明显，土地经营纯收益6.3亿元，增长95%；公开拍卖出租车经营权、绿地养护权、街路清扫保洁权等，实现收益1.3亿元，节省资金3.5亿元。生态建设扎实推进，新建完善生态园34个，新建城市绿地108万平方米，建成区绿化覆盖率达35.6%。完成湿地和生态补水4.2亿立方米，植树造林17.5万亩。

　　（四）改革开放不断深化。稳妥推进418户企业下岗职工失业救济向失业保险并轨，并轨人员达到4.9万人，发放经济补偿金3.7亿元，解决了原国有企业改革遗留下的人员包袱和社会负担问题。推进事业单位改革，实行职称评聘分开和新增人员凡进必考制度；进一步规范编制管理，清理取消各类临时性议事协调机构9个。深化行政审批制度改革，审批事项由1048项减少到503项，取消行政收费212项，废止规范性文件106件。理顺教育管理体制，接收企办125所学校、9个教育管理机构、教职工1.5万人。推行教育系统人事制度改革，实行校长聘任制和教职工全员聘用制。深化警务体制改革，实行扁平化管理，充实了基层警力，实现了网格化布局，命案攻坚、打击刑事犯罪等20个方面工作位居全省第一。推进粮食企业改革，稳妥解决粮食企业历史挂账和拖欠职工债务等问题。深化商业银行股份制改造，资产总额达到74.7亿元，比年初增加22亿元，赢利7600万元。农村信用社和国有资产运营体制改革取得明显进展。招商引资富有成效，引进亿元以上项目45个，实际到位资金103亿元。进出口贸易总额达到2.7亿美元，实际利用外资3081万美元，分别增长119.2%和28%。

　　（五）社会事业全面进步。科技事业，搭建5个中试平台，组建7个生产力促进中心，建立14家企业研发中心，申请专利技术490项，科技创新体系不断完善。教育事业，高校招生1.7万人，全市在校生达到5.7万人；高考二表进段人数首次突破6000人，万人口升学比例继续保持全省第一；改造34所农村d级危房校舍，铺设农村通校路、校园甬路，33所农村寄宿制学校实现锅炉取暖，为510所农村学校配备计算机，教育满意度全国排名第六。卫生事业，市人民医院、龙南医院分别成为哈尔滨医科大学、齐齐哈尔医学院附属医院；社区卫生服务机构达到138家，国家级社区卫生服务示范区数量全国第一；市县疾控中心基本建成，在全省首家实现乡镇卫生院疫情网上直报；全面推行农村新型合作医疗，农村人口参合率达70%，公共医疗服务水平实现新的提高。文体事业，大型多媒体话剧《铁人轶事》在全国公演，成功举办第九届地企联合运动会，群众性文体活动广泛开展。

　　（六）公共秩序和谐稳定。政府建设得到加强。坚持依法和民主行政，认真执行人大及其常委会决议、决定，自觉接受人大、政协和社会监督，办理人民代表议案、政协委员提案281件。出台了市政府工作规则、重大行政决策听证、行政问责、违反决策程序责任追究、新闻发言人等10多项制度办法。坚持领导信访接待制度，组建市长公开电话受理中心，政府与各界沟通渠道进一步拓宽。社区建设成效明显，活动场所、办公条件进一步改善，便民服务网点达到8238家。社会治安综合治理不断强化，查处治安案件3.2万起，破获刑事案件1.4万起，破案率达到61.8%，有力打击了各种违法犯罪活动。开展整治油田及周边治安专项斗争，成立油田公安分局，打击侵油犯罪活动，切实维护了油田正常生产生活秩序。建立安全防范例会制度，严格实施安全生产责任制，实现公共安全、社会稳定，人民群众安居乐业。大庆被评为全国社会治安综合治理优秀城市。

　　此外，工商税务、统计物价、邮政通讯、新闻出版、民族宗教、文史档案、外事侨务、司法、人防、计划生育、国防教育等项工作，也都取得了新的成绩。

　　回首“十五”发展历程，总结过去一年工作，我们切身感受到，所有成就的取得，是市委带领全市人民解放思想、努力拼搏的结果，是中直石油石化大企业积极开拓、合力共创的结果，是市人大、市政协监督支持，各民主党派、人民团体及老领导、老同志积极帮助的结果。在这里，我代表市政府，感谢驻庆中省直各单位，为大庆发展建设做出的重要贡献；感谢各位人大代表、政协委员，为政府工作提出的真言良策；感谢各条战线广大干部群众，为完成“十五”阶段性目标付出的艰苦努力。全体政府公务人员将以此为激励，奋发有为，开拓进取。

　　回首“十五”发展历程，总结过去一年工作，我们深刻体会到，全面贯彻落实科学发展观，推动全市经济社会又快又好发展，必须坚持发展第一要务，立足创建“百年油田”，不断壮大接续产业；必须坚持改善民生，努力维护好、实现好、发展好人民群众的根本利益，使全市人民共享改革与发展成果；必须坚持走自主创新之路，建设科技促发展、教育图振兴、人才兴大庆的创新型城市；必须坚持深化改革、扩大开放，营造开放竞争、充满活力、适应市场经济发展要求的体制环境；必须坚持统筹城市与农村、经济与社会、人与自然协调发展，构建和谐社会；必须坚持地企合作，做到责任共担、发展共促、稳定共抓、城市共建。这些，既是以往政府工作实践的切实体会，也是今年乃至今后一个时期应当坚持的指导方针。

　　回首“十五”发展历程，总结过去一年工作，我们也清醒地看到，实现大庆经济社会科学发展，仍然面临不少问题和困难。主要是，支撑可持续发展的产业基础不够牢固，接续产业相对弱小，具有较强市场竞争力的优势企业和品牌产品还比较少；经济外向度较低，招商引资层次不高；“三农”问题仍然突出，农业和农村经济的实力不强、后劲不足，促进农民增收的长效机制尚未形成；传统服务业升级、自主创新等方面还没有完全破题；部分群众生活困难，城乡就业压力较大，等等。政府工作也存在缺点和不足，主要是，职能转换存在差距，市场监管、社会管理和公共服务有待加强，办事效率和依法行政能力有待提高。这些问题，我们一定认真对待，统筹考虑，着力解决。

　　二、“十一五”规划目标和2006年主要工作

　　“十一五”是大庆实现可持续发展和全面建设小康社会承前启后的关键时期。按照市委六届四次全会提出的在全省率先基本实现现代化的总体要求，到2010年，全市经济社会发展的主要目标是：地区生产总值年均增长8%、人均生产总值超过7万元；非油经济占全市经济比重达到50%左右；万元生产总值综合能耗比“十五”期末降低20%以上；科技对经济增长的贡献率达到50%；开放型经济体系初步确立；现代都市框架基本形成；社会主义新农村建设迈上新台阶；城乡居民物质文化生活质量显著提高，城镇登记失业率控制在4.5%以内，城镇社会保险覆盖率达到90%，人口自然增长率控制在5.6‰以下，城市居民人均可支配收入达到18000元，农民人均纯收入达到5000元，建成全国文明城市。主要任务是：

　　———创建“百年油田”，发展壮大非油工业。坚持原油硬稳定、天然气快发展，做大做强化工产业，培育壮大新的接续产业。油气当量保持在4200万吨以上，把石化工业做成超千亿元的产业，同时推动乳业、农产品加工、新能源、纺织和皮革、机械制造和电子信息、新材料和橡胶等重点产业规模超百亿元，形成石油、石化、地方工业三大支柱。到2010年，实现工业增加值1460亿元以上，年均增长7%以上。

　　———建设社会主义新农村，增强县域经济实力。按照“生产发展、生活宽裕、乡风文明、村容整洁、管理民主”的要求，继续调整农村经济结构，实施“农转牧”战略，畜牧业增加值占农业比重达到60%以上；加强基础设施建设，完成500万亩抗旱保收田建设任务；发展农村公共事业，完成“十一五”规划确定的道路、通讯、电网、饮水工程建设目标，全面实行农村合作医疗。全市80%的村达到社会主义新农村建设基本标准。到2010年，第一产业实现增加值80亿元以上，年均增长13%以上。

　　———改造提升传统服务业，加快发展第三产业。按照市场化、产业化、社会化的方向，培育发展金融保险业、现代物流业、信息咨询业；壮大提升流通业，打造东、西两大商业中心区，加速发展旅游业，叫响“绿色油化之都”、“天然百湖之城”；大力发展中介服务组织，提高社区服务产业化水平。到2010年，第三产业实现增加值400亿元，年均增长17%。

　　———加强基础设施建设，打造现代都市框架。完善城市路桥、通讯、供电、供水、供热、供气等基础设施，提升城市品位，改善人居环境。继续实施东移北扩、中联西拓，构筑以东、西主城区为核心，以红岗、大同、林源为重要组团，以中心小城镇为支点的多层次城市体系。

　　———增强自主创新能力，建设创新型大庆。把创新作为发展的主题，把增强自主创新能力作为调整产业结构、转变增长方式的中心环节。推进科教兴市，整合优化科技资源配置，搞好关键技术开发，力争在石油石化和生物、新材料、农产品深加工、电子信息、节能环保、先进制造等领域，形成一批拥有自主知识产权的核心技术，推动“大庆制造”向“大庆创造”转变。发挥大学、科研机构作用，建设创新人才队伍，搞好产学研联合，为大庆经济社会发展提供智力支撑和人才保证。

　　———发展循环经济，创建资源节约型和环境友好型社会。坚持开发节约并重、节约优先，以减量化、再利用、资源化为原则，以低消耗、低排放、高效率为特征，发展节地、节水、节能、节材的节约型经济，推进节能降耗，促进资源循环利用。大力加强生态市建设，综合整治水环境，预防控制污染，保护湿地、草原，把大庆建成生态型园林城市。

　　———继续深化改革、扩大对内对外开放。加快行政管理体制改革，分类实施事业单位改革，继续深化国有企业改革，积极推进财政、金融、投资等体制改革，建立比较完善的适应市场经济要求的管理体制和运行机制。扩大招商引资，发展外经外贸，培育壮大外向型骨干企业，提高经济外向度。

　　———建设和谐大庆，繁荣各项社会事业。大力发展教育、卫生、文化、体育、福利等社会事业，提高城乡居民物质文化生活水平。扩大社会就业，完善社保体系，改善困难群众生活。加强精神文明和民主法制建设，妥善处理人民内部矛盾，维护公民合法权益，确保社会秩序和谐稳定。

　　各位代表，2006年，是实施“十一五”规划的起步之年，也是推动大庆迈向科学发展新阶段的关键之年。政府工作要以邓小平理论和“三个代表”重要思想为指导，用科学发展观统领经济社会发展全局，认真贯彻市委六届四次全会精神，坚持“落实、提升、创新、突破”的工作基调，以发展经济改善民生为主线，抓住大事，办好实事，破解难事，努力实现经济社会又快又好发展。主要预期指标是：地区生产总值增长8%、力争达到9%，万元生产总值综合能耗下降4%，地方财政总收入比上年预算增长15.2%，固定资产投资增长8%以上，城市居民人均可支配收入达到14350元，农民人均纯收入达到3000元。重点做好以下六个方面工作。

　　（一）推动城乡经济快速发展。

　　———加快发展园区化工业经济。坚持工业向园区集中、产业向集群发展，做大、做强、做专各类工业园区，全市工业增加值力争完成1290亿元，增长8.5%。新建续建150个项目。在支持油气产业稳步发展的同时，进一步壮大石化产业，配合大企业争取新建60万吨乙烯、30万吨聚丙烯和100万吨复合肥扩建等项目，推进6.5万吨1,4-丁二醇、10万吨苯乙烯、5万吨甲乙酮等新建项目开工，抓好20万吨醋酸、50万吨ddc、10万吨干气制乙苯等续建项目建设。扎实推进地方工业“百项工程”，重点抓好30亿只药用玻璃制品、300万条半钢子午胎等新建续建项目，确保地方工业增长35%，力争达到40%。提升19个园区功能。对高新区“一区三园”、轻纺、皮革、大豆、精细化工等园区，加大基础设施建设力度，完善园区功能，启动建设西城省级工业园。加快哈大齐工业走廊大庆项目区建设，新建项目100项。推进科技创新。用好政府科技风险资金，选择市场前景好、科技含量高的科研成果，进行中试放大，加快产业化步伐。搞好技术引进、消化和再创新，争取国家、省级创新项目20项以上，努力培育一批高新技术企业，加快壮大高新技术产业。发挥好生产力促进中心作用，建设市、县（区）两级科技市场，完善高新区创业服务中心和软件园等孵化器，为企业技术创新提供服务支撑。

　　———积极推进社会主义新农村建设。坚持以工业化的思维谋划农业和农村工作，着力发展工业主导型农村经济。继续推进农业结构调整。鼓励发展经济作物和饲草饲料产业，大力发展畜牧业，完善资金支持、科技推广、繁育改良、防疫灭病等服务体系。搞好农业产业化经营。围绕乳品、生猪、大鹅、玉米、蔬菜等10个产业，做强龙头，做优基地，用龙头企业群带动形成产业群。重点培育完善农产品专业批发市场、市中心农贸市场、绿色食品展销中心、县域农产品集散地、农产品出口基地。发展新型农村合作经济组织，建设农民经纪人队伍，完善农村社会化服务体系。加强农业基础建设。新打水源井1211眼，新增配套设备2235台套，建设抗旱保收田67万亩。继续加强南引水库二期、东城水库等大型水源基础设施建设，抓好小流域治理。实施标准示范工程。全面推进“六吨奶”工程，奶牛单产平均达到5.5吨。组建60个农机作业合作社，进一步提高农业生产机械化水平。建设标准化示范基地，全市特色绿色基地面积发展到300万亩以上，饲草饲料基地面积达到120万亩。实施良种化工程，引进优良品种，提高良种普及率。抓好社会主义新农村建设试点。完成新农村建设规划，分梯次推进，195个村达到基本建设标准。着力搞好以解决吃水难、出行难为重点的公共服务设施建设，以改善村容屯貌为目标的农村环境综合整治，以文明、科学、健康为内容的农村精神文明创建，以村民自治为特色的农村民主建设。认真开展农业普查。增加市本级财政对农村的资金投入。

　　———加快传统服务业提档升级。建设中心商业区。整合商业资源，提升业态水平，完善建设西城区和东城区两大中心商业区。重点培育和发展汽车流通市场，家电、电子信息和通讯产品消费市场，促进现代建材装饰和家居用品消费市场的发展。积极引进沃尔玛等国内外知名企业，发展壮大庆客隆等连锁龙头企业。积极培育餐饮娱乐、文化艺术等特色消费街区，营造繁荣活跃的商贸环境。发展物流产业。整合物流资源，培育物流市场，重点建设大庆石油物资物流园区、大庆港桥物流园区，积极发展第三方物流业。提升旅游业水平。实施以石油文化游为龙头、温泉度假游为特色、湿地休闲游为亮点、民俗风情游为辅助的“四季旅游”工程，开辟市区通往景区旅游客运专线，开发客源市场，建设精品景点，繁荣旅游经济。推动金融业开放发展。争取引进招商银行等金融机构，促进金融业竞争发展。强化企业诚信建设，优化金融发展环境。协调金融部门积极向上争取政策，有效提高金融业对全市经济的支持力度。

　　（二）努力提高人民生活水平。

　　———千方百计扩大就业和再就业。落实促进就业再就业的各项政策，实现新增就业3万人，领证人员再就业2.3万人。开发就业岗位，加快劳动密集型产业发展，增加就业机会；加大公益岗位开发，安置困难群体。加强专业培训，搞好市场对接，在全国扩大电焊、汽车维修、机械加工等劳动力品牌影响。发展劳务经济，提高农村劳动力转移的组织化水平，扩大转移总量，增加劳务收益。强化就业服务，实行创业培训与小额贷款相结合，降低创业门槛，增加创业机会。加强劳动力市场管理，整合人力资源市场，加大劳动保障监察力度，维护劳动者合法权益。关注新失业群体，重视大中专毕业生和复转军人的就业指导与服务。努力消灭“零就业”家庭，让每个家庭有人就业。

　　———健全完善社会保障体系。完善综合救助体系，提高低保水平。采取多元化救助形式，丰富救助内容，扩大救助范围；在城市启动实施低保群众重大疾病救助，切实解决低保家庭就医负担重问题；帮助残疾人就业，改善残疾人生活质量。继续加大社保扩面力度，完善社保机制。将应保未保的混合所有制企业、非公有制经济组织从业人员等纳入社保范围；出台无地、失地农民参加养老保险办法；启动四县工伤保险和生育保险市级统筹，建立职工家属医疗保险社会统筹、机关事业单位职工工伤和生育保险制度。提高企业职工最低工资标准。

　　———继续抓好社区建设。完善社区基础设施，解决47个社区无办公场所和活动场所问题，在新建住宅区配套建设社区基础设施。强化社区功能，引导行业协会、中介组织和个体私营业者发展社区服务业，搞好社区文化、治安、环境建设。调整完善社区工作机构和职能，健全具有就业、低保、维权、助老等多项职能的工作体系。建立“96760”社区服务呼叫中心，为社区居民提供更加优质的服务。

　　———切实保护农民利益。认真落实“一免两补”等惠农政策，有效减轻农民经济负担。加强农资市场秩序和质量监管，实施农资最高限价，稳定农资价格水平。推进整村扶贫开发工程，加快60个重点村、7200户脱贫步伐。新建农村饮水安全工程90处，解决4.7万农村人口饮水难问题。启动实施农村居民最低生活保障制度，提高“五保户”供养水平，确保无劳动能力、无收入来源困难农户的衣食供应。采取措施救助农村贫困大学生。对农村义务教育阶段家庭困难的学生，免除杂费、书本费，补助生活费。组建农民工维权机构，依法保护农民工特别是外出务工农民权益。

　　———努力改善群众生活环境。加快城中村和棚户区改造，推进富强村和龙凤澳龙、铁东平房区拆迁建设。继续实施新村7区、万宝小区、乘风8区等小区环境改造，提升老居住区功能。实施萨尔图区、龙凤区部分老城区饮水解困工程，建设临时供水站。将29所敬老院合并改造为11所。以食品药品为重点，整顿规范市场秩序，严厉打击制假售假、欺行霸市、哄抬物价等侵害消费者利益的行为。加强食品质量技术监督，建立粮油质量监管中心，创办“放心粮油”综合批发市场。加强价格调节基金征收管理，平抑居民液化气价格，开展教育收费、医药价格专项检查和整治，降低群众生活成本。抓好城市数字电视普及推广，力争新增用户12万户。推进“村村通广播电视”工程，争取4万户以上农民看上数字电视。加快天然气入户进程，实现3.1万户住宅开通使用。兑现公务员津贴补贴，提高公务员工资标准。

　　（三）抓好改革开放和招商引资。

　　———继续深化各项改革。加快推进事业单位改革。按照政事分开的原则，对事业单位重新界定分类，对不参照公务员管理的事业单位，普遍推行全员聘用制。进一步完善教育管理体制，力争完成油田教育中心、石化教育中心所属学校人事制度改革。加快建设公共财政体系，逐步提高各类公共服务的支出比重，强化综合预算管理，完善财政投资评审和集中收付制度。改革投资管理体制，实施非政府投资项目核准制和备案制，推行项目代建制、招投标制和项目稽察制，提高政府投资效益。继续深化农村综合配套改革，巩固扩大农村税费改革成果。加快粮食企业和原市建一、二公司改革。大力扶持非公有制经济发展，着力在申报政府计划项目、土地使用、开展进出口业务、科技创新、资质评定、融资扶持等方面，为民营企业创造便利条件。

　　———促进招商引资战略升级。根据我市产业发展方向，着力在引进大项目、科技含量高的项目、外资项目上下功夫。做好载体招商，充分发挥哈大齐工业走廊大庆项目区和现有专业园区的载体功能，实行专业招商，做大园区，做强产业。做好论坛招商，充分利用各级行业协会的影响力，围绕产业和园区举办论坛活动，邀请知名专家和企业家，宣传大庆、投资大庆。做好重点招商，充分整合资源优势，盯住国内外大企业，特别是世界500强企业，引进战略投资者，建设产业龙头，带动相关产业整体发展。做好选商招商，已经成形的专业园区，重点引进实力型企业、关键的配套型企业、科技领先型企业。在招商引资中，注重综合考察投资强度、环境保护、吸纳就业、产品品牌和税费贡献，节约利用土地。

　　———提高对外经贸水平。全力做好出口加工区报批工作，研究推进内陆港和保税仓库建设，严格建设水平和入驻标准，打造对外经贸窗口。支持石油国际工程公司等有外贸潜力的企业做大做强，努力打造一批超亿美元的大型龙头企业。在俄开发建设大庆商品展销中心，拓宽对俄经贸通道。出台扶持龙头出口企业的暂行办法、市内企业在境外开发设立商品展销窗口暂行办法等支持政策，推动外向型经济快速发展。

　　———进一步优化经济发展环境。建设利于企业落地生产的产业生态环境，加大19个工业园区基础建设投入，完善园区水、电、气、热、讯、路等配套设施，降低企业入驻门槛，建设专业标准厂房，使企业设备进驻就能启动生产，比照哈大齐工业走廊优惠政策，全面减免各类行政规费，建设特色园区、品牌园区。搞活利于企业发展壮大的融资环境，发挥担保中心作用，做大做强商业银行，建立风险投资机构，提高金融业服务水平。打造利于企业轻松办事的优质服务环境，推行“政务超市”服务模式，建立方便快捷的政务流程，重大项目和重点企业一事一议、特事特办，进一步提高行政服务效率。

　　（四）切实搞好城乡建设管理。

　　———抓好重点项目建设。用打造大都市格局的思维规划项目，用完善城市功能的标准确定项目，用协调和经营的手法运作项目，新建续建教育文化中心、青少年活动中心、会议中心、行政服务中心、东安市场改造等工程389项，总建筑面积506万平方米。统筹东、西城区协调发展，不断完善西城区功能，在让胡路区新建铁路客运总站，建设中央大街立交桥，打通西苑街，拓宽西干线，扩建中三路，完善让杜路，迁出后四厂，改造庆虹桥，实施让通线“平改立”，支持大企业建设大庆石油博物馆，进一步繁荣西城区、提升西城区。加快五湖新区项目开发，做好环城路西段、兰德湖区域整治、北国之春绿色温泉城、东湖水厂扩建等项目论证和招商，推进湖滨教师花园二期和靓湖国际花园住宅开发，完成11条高压线路改造，努力打造具有北国水乡特色的生态新区、功能新区和景观新区。进一步完善交通基础设施，加快飞机场立项建设，继续改造萨大路，续建g015大庆绕行线，完成经九街延伸，做好大广高速大庆段建设前期工作。新建通乡村公路847.6公里，在全省提前4年率先实现“村村通”。

　　———提升城市管理水平。开展城市环境综合整治年活动，重点推进净、绿、亮、美、畅工程。加大环境卫生治理力度，增加垃圾排放场、公厕数量，消灭卫生死角死面。新建7个生态园，新建城市绿地580万平方米，建成区绿化覆盖率达到36.5%。实行绿色照明、限时亮化，抓好城市街路、小区照明，搞好楼宇和围湖亮化，新增10条亮化街路。搞好广场、小区等重点区域重大节日的美化活动，扮靓城市形象，营造喜庆气氛。整顿规范交通秩序，严厉打击非法营运。加强建筑市场整顿，创建开放有序、公平竞争的良好环境。巩固“创模”成果，确保我市顺利通过全国环保模范城复检。探索实施数字化城市管理，建立城市管理“大庆市网络数据中心”，创建数字档案，提升数字移动通讯水平，加快实现城市管理现代化。

　　———整合经营城市资源。以经营土地为核心，以项目建设为载体，深入探索“协调换投入、资源换资金、无形换有形”的城市经营模式。严格土地管理，坚持统一规划、连片开发、总量控制、招标拍卖、一级垄断、净地出售，对重点开发区域控制供地总量，对建设用地实行不饱和供应，计划出让五湖新区和红旗地区建设用地500公顷，收储土地490公顷，完善地价调控机制，确保增值收益。搞好出租车经营权、高空广告空间使用权，以及街路冠名权、清扫保洁权等方面的经营，盘活人防车库等存量资产，进一步增加经营收益。探索运用市场化方式建设经营文化设施，逐步实现自主经营，自我发展。

　　———加强生态环境建设。继续实施以造林为重点的植树绿化工程，抓好以“堵西北风口”为重点的防沙治沙造林工程及村屯绿化，全年造林15万亩，森林覆盖率达到10%。加大草原管理力度，扩大禁牧成果，推广封区育草。抓好环境保护，实行环境准入制度，对所有建设项目进行规划建设环评。强化工业污染防治，对重点污染源采取强制审核、排污许可、挂牌整治、限期治理，实现达标排放，加大市区内环境噪音和污染防控力度。抓好果午泡、东风泡、明湖、新潮湖、滨洲湖和黎明河“五湖一河”环境治理。

　　（五）积极促进社会和谐。

　　———加强精神文明建设。继续深入开展全国文明城市、全国卫生城市创建活动，努力塑造文明整洁的城市形象。成立大庆精神、铁人精神研究会，弘扬发展大庆创业文化，塑造新时期大庆人的崭新形象。征集宣传以回忆大会战、展现新大庆为主要题材的文化艺术精品，编辑出版《大庆文艺精品丛书》，进一步提高大庆的知名度和影响力。宣传贯彻《公民道德建设实施纲要》，加强和改进未成年人思想道德建设和大学生思想政治工作，全面提升市民文明素质。搞好农村文化站、城市社区和广场文化设施建设，举办系列文化活动，繁荣文化事业。

　　———促进教育平衡协调发展。解决就学难问题，调整教育布局。在靓湖国际花园、万宝3区、丽水华城规划建设九年一贯制学校。撤并农村规模小、效益低的办学点，在中心乡镇集中建设寄宿制学校。改善办学条件，改造聋哑学校、大庆中学、石油高中等学校，继续抓好农村新产生的d级危房校舍维修，完成剩余的26所农村寄宿制学校锅炉改造。为510所微机空白校配备计算机，实现“校校通”。满足就业需求，改造建设大同区职业教育学校，将建设中专迁入广播电视大学，加快职业学校实习基地及技能鉴定中心建设，打造全国知名的职业教育基地。

　　———提高城乡公共卫生服务水平。市级综合医院提升整体医疗水平，普通医院发展特色专科，加强重点专科建设，加强对外交流与合作，创造高水平的医疗服务。建设和普及标准化社区卫生机构，扩大服务网络。实施医疗质量信息公示制度，实行手术费限价，抓好“平价医院”和“平价药店”建设，减轻群众医疗负担。完成市中医院异地新建的规划论证和启动建设，抓好四医院维修改造。在全市农村深入推行新型合作医疗制度，农民参合率力争达到85%。提升实施卫生支农“七个一”工程，提高乡镇卫生院的医疗服务水平。

　　———加强社会管理。健全完善政府负责、各界协同、公众参与的社会管理格局。在科学完善《大庆市自然灾害和公共安全突发事件防控应急预案》的基础上，建立14个子预案，进一步健全社会预警体系和应急救援机制，提高处置突发事件的能力，强化全民对公共安全的防控应急意识。理顺街道、社区、物业、供热等方面之间的关系，完善社区业主委员会职权，提高城镇居民自治管理水平。扩大村级民主选举、民主决策、民主管理和民主监督，健全基层社会管理体制，促进农村社会协调发展。规范各类民间组织行为，发挥其在反映民意、调处矛盾等方面的积极作用，加强政府与民众、企业及社会各界的联系沟通，营造和谐文明的社会氛围。

　　———做好安全稳定工作。继续深化警务体制改革，全力推进科技强警，加快公安综合信息系统建设，尽快实现“110、119、122”三台合一。加强基层防控网络建设，力争80%的社区建立平安网络。搞好社会治安综合治理，对重点区域、重点部位、重点场所强化管理，开展专项打击和整治行动，保持严打高压态势。严厉打击黑恶势力、黄赌毒等违法犯罪活动，坚决打击邪教组织，维护社会稳定和政治安定。认真抓好信访工作，综合治理上访秩序，依法、及时、就地解决人民群众反映的问题，从源头上预防和化解社会矛盾。落实安全生产责任制，防止重特大生产事故和交通事故发生，建设平安大庆。

　　（六）切实为石油石化企业服务。

　　重点抓好10件事：

　　一是协调解决占用农田打井用地问题，推进拆迁占压油田管线建筑，完成中五路、胜利村等地区22.8万平方米拆除任务，为油田开发生产清理空间。

　　二是整治油田生产治安秩序，严厉打击盗抢原油和大企业物资等犯罪活动，确保安全生产。

　　三是努力改善西城区环境，抓好红旗村拆迁建设，畅通西城交通，协助后四厂拆迁，创造良好的生活环境。

　　四是联合实施天然气入户工程，加快推动民用天然气产业开发经营。

　　五是共同维护社会稳定，协助解决企业职工信访问题，支持企业搞好安全生产防控。

　　六是积极创造就业岗位，通过共同上项目、建企业等方式，努力为下岗职工创造更多的就业机会。

　　七是地企携手向上争取政策、资金和项目。

　　八是联手活化大企业存量资产，联合研究开发大项目，协调大企业加大对地方项目投资力度，联合启动建设西城省级工业园，继续开发利用外围油田，进一步增强地企经济融合度。

　　九是探索合作开发大企业生产生活消费市场的新途径，整合区域生产要素，加快建立对接大企业的配套生产和物流基地。

　　十是继续坚持地企沟通协调制度，定期召开地企高层领导联席会议，做到大事一起定、工作合力抓。

　　各位代表，创建百年油田，构建战略新高，共建和谐社会，推动大庆迈上科学发展新阶段，全市各级政府肩负着光荣而艰巨的历史使命。我们要始终抓住发展经济、改善民生这条主线，始终坚持以人为本、执政为民的理念，加倍努力建设学习型、创新型、法治型、服务型、廉洁型政府。

　　一是深入推进政府职能转变。在继续完善经济调节、市场监管的同时，注重加强社会管理和公共服务。把政府工作的重点、政策支持的重点、财力保障的重点，进一步向社会事业发展倾斜，向公共基础设施建设倾斜，向生态建设和环境保护倾斜，向扩大就业、完善社保体系、改善困难群众生活倾斜。

　　二是坚持依法行政。贯彻落实国务院《全面推行依法行政实施纲要》，提高行政执法效率和水平。认真执行人大及其常委会决议，自觉接受监督。坚持政府重大事项报告制度，遵守政府组成人员向人大述职制度，自觉接受评议，认真及时整改。加强与政协及各民主党派、工商联和无党派人士的联系。认真办理人大议案、建议及政协提案，提高承办质量和水平。坚持科学民主决策，完善重大决策集体讨论、专家咨询、公示和听证等项制度，实施群众代表和法律顾问列席政府常务会、新闻发布、人民建议征集等项制度，建立民意评价政府工作机制。

　　三是拓宽政务公开范围。推进透明政务，打造阳光政府。完善便民利民的公开渠道，清理市本级、市政府各部门行政权力，清理公用事业单位服务项目，并向社会公开。重新审定行政权力的运行流程，建立规范、科学、高效的政府工作运行体系。公开政府机关的职责范围、办事程序、重大决策和办事结果。推行公用事业单位办事公开制度，对涉及学校、医院、供水、燃气、交通等与群众利益密切相关的公用事业单位，公开职责权限、办事流程、收费依据、经营状况等等。

　　四是努力提高行政效能。深化行政审批制度改革，对全市行政审批和行政收费项目进行再次清理，简化审批程序，减少审批和收费事项，依法规范行政机关执法职责权限。建立行政服务中心，实行“一门受理、全程跟踪、一站式服务”。加大行政效能监察力度，禁止部门和单位以各种名义干扰企业生产经营活动。实施行政效益审计，建立政府决策后绩效评估机制和经济社会发展综合评价体系。加快推进电子政务。建设网上办公系统，实行公文网上流转。建设网上咨询系统，满足企业和群众咨询需要。建设网上审批系统，探索行政许可和行政审批事项上网运行。

　　五是公正廉洁从严理政。认真执行廉政建设各项规定，完善相关制度，加强对财政资金使用、专项资金管理、工程招投标、土地批租、人事管理、行政执法等重点领域的监管。实施行政执法责任制和行政责任追究制，对行政不作为和乱作为、有令不行、推诿扯皮，影响行政执行力的行为，严肃追究责任。加强审计监督，反对浪费铺张，严肃财经纪律，建设人民信任政府。

　　六是改进工作推进方式。实行三维分工和考核，政府各级领导干部既要负责分管战线工作，又要承担安全稳定和招商引资包项目工作，并以此为依据，综合考评业绩。

　　各位代表，大庆发展已有了一个好的基础、好的氛围、好的势头。让我们在市委的坚强领导下，发扬大庆精神和铁人精神，紧紧依靠全市各族人民，解放思想，求真务实，同心同德，开拓创新，为构建大庆全面发展战略新高地而努力奋斗！
